# A Novel Automate Python Edge-to-Edge: From Automated Generation on Cloud to User Application Deployment on Edge of Deep Neural Networks for Low Power IoT Systems FPGA-Based Acceleration

**DOI:** 10.3390/s21186050

**Published:** 2021-09-09

**Authors:** Tarek Belabed, Vitor Ramos Gomes da Silva, Alexandre Quenon, Carlos Valderamma, Chokri Souani

**Affiliations:** 1 Electronics and Microelectronics Unit (SEMi), University of Mons, 7000 Mons, Belgium; vitor.ramosgomesdasilva@umons.ac.be (V.R.G.d.S.); alexandre.quenon@umons.ac.be (A.Q.); carlosalberto.valderramasakuyama@umons.ac.be (C.V.); 2 Ecole Nationale d’Ingénieurs de Sousse, Université de Sousse, Sousse 4000, Tunisia; 3 Laboratoire de Microélectronique et Instrumentation, Faculté des Sciences de Monastir, Université de Monastir, Monastir 5019, Tunisia; 4 Institut Supérieur des Sciences Appliquées et de Technologie de Sousse, Université de Sousse, Sousse 4003, Tunisia; Chokri.souani@issatso.u-sousse.tn

**Keywords:** cloud computing, deep neural networks (DNNs), edge computing, field programmable gate array (FPGA), hardware acceleration, high-level synthesis (HLS) tools, internet of things (IoT), low-power, low-cost, Python framework

## Abstract

Deep Neural Networks (DNNs) deployment for IoT Edge applications requires strong skills in hardware and software. In this paper, a novel design framework fully automated for Edge applications is proposed to perform such a deployment on System-on-Chips. Based on a high-level Python interface that mimics the leading Deep Learning software frameworks, it offers an easy way to implement a hardware-accelerated DNN on an FPGA. To do this, our design methodology covers the three main phases: (a) customization: where the user specifies the optimizations needed on each DNN layer, (b) generation: the framework generates on the Cloud the necessary binaries for both FPGA and software parts, and (c) deployment: the SoC on the Edge receives the resulting files serving to program the FPGA and related Python libraries for user applications. Among the study cases, an optimized DNN for the MNIST database can speed up more than 60× a software version on the ZYNQ 7020 SoC and still consume less than 0.43 W. A comparison with the state-of-the-art frameworks demonstrates that our methodology offers the best trade-off between throughput, power consumption, and system cost.

## 1. Introduction

Over the last few decades, both Artificial Intelligence (AI) and the Internet of Things (IoT) have seen considerable development and adoption in numerous domains [1,2,3,4]. Although they were not originally meant to be merged, some specific applications require the accuracy and performance offered by AI algorithms, specifically by Deep Neural Networks (DNN), while being constrained by typical IoT considerations, such as the low power consumption [5]. This is for example the case of edge computing, with the local acquisition and processing of peripheral data. Consequently, particular challenges due to the deployment of DNNs to the edge have arisen [5,6,7]. The main technical difficulties originate (1) from the high computing demand of the DNN-related algorithms, whereas the edge and IoT nodes generally offer a limited computational power, and (2) from the usually high power consumption requirement, also not compatible with the target deployment platform. To solve these problems, dedicated embedded systems have been proposed: using reconfigurable circuits, the Field Programmable Gate Arrays (FPGA), and System-on-Chips (SoC), a complete system embedded on a single chip, which specifically targets the deployment of DNN for edge computing and the Internet of Things. Nevertheless, a major challenge remains the design flow which requires a know-how combining hardware design on FPGA with neural network architectures, to be able to build the appropriate network for the chosen application [8]. An additional challenge is to make the use of very distinct design and application flows compatible and transparent. This challenge has been partly mitigated by the appearance of High-Level Synthesis (HLS) tools that help to divide the tasks between the CPU and the FPGA in an optimized way, performing the so-called hardware acceleration.

In this paper, we propose a solution that simplifies the design and deployment of a deep neural network architecture to the edge: a fully automated framework that provides a Python interface to create and optimize a DNN, run the synthesis on the Cloud, and deploy the resulting network directly on the IoT user application node, i.e., the Edge platform. Compared to other works proposing a configurable black box, development and optimization of results are accessible, revealing the impact of implementation choices in a simple, concise, and controlled way. The framework, based on the co-design approach, consists of simultaneously designing the software and hardware parts of the neural network architecture to improve the quality and consistency of the deployed solution.

In addition, by offering a Python interface while relying on low-level hardware design, it brings together hardware designs and software development skills, which helps to fasten and improve the design process [9,10]. The detailed work presented in this article is considered as a continuation of our published work [11], where we have presented in depth the optimization of the hardware architecture as well as a model to estimate the performance that can be achieved depending on the level of optimization. Many figures have been provided for space exploration depending on the level of optimization and the size of the DNN topology. In this paper, we go forward to provide a new full Edge-to-Edge automated generation environment. In summary, contributions of this work are listed bellow:

- A Python interface allows the user to customize the DNN implementation at the Edge based on the target platform’s limited hardware resources and the desired performance.
- Balancing the optimization techniques as well as the interface protocols of each IP in order to meet design-entry requirements and FPGA restrictions. For that, the customization process starts from a C++ template that has several default input parameters to optimize and encapsulate the IP layer to be generated. The generated IPs will be stacked to build such DNN topology.
- Once the customization is complete, the framework generates a TCL file that runs the pre-installed tools on the host server (or a commercial cloud such as AWS or Google Cloud) where all the necessary HLS development tools are pre-installed.
- The IP hardware (bitstream file) and the cloud-generated software library can easily be deployed to the Edge from the user application. The generated DNN can also be uploaded to a website platform (e.g., Github, the AWS Marketplace) to be shared with other users.
- Thanks to our new design flow, the user can easily customize, optimize, generate, and use DNN models on the Edge without needing to master hardware development tools, as is the case with almost all cutting-edge works. To our knowledge, this is the first automated environment that develops and deploys DNN architectures based on end-to-end FPGA acceleration.

This paper is organized as follows: Section 2 reviews the state-of-the-art hardware optimization techniques as well as frameworks that offer co-design and methodologies for the deployment of neural networks to the edge. Section 3 presents a general overview of the proposed framework, whereas Section 4, Section 5 and Section 6 explain the implementation details of the DNN configuration (on the Edge), the generation of the FPGA architecture (on the Cloud), and the communications between the Cloud and the Edge, respectively. Section 7 describes and compares the results with the state of the art. Finally, Section 8 draws the conclusions.

## 2. Related Works

Many works in the literature proposing reconfigurable computing to enhance DNN algorithms have demonstrated a speed increase when compared to CPU and GPU implementations [12,13,14,15]. The latter is commonly employed to speed up DNN topologies because it provides better outcomes in terms of pure processing performance. However, regarding the processing performance for the power consumption ratio, a high-throughput GPU is proved to be inefficient. Consequently, GPUs are not widely used for embedded edge computing [16]. On the other hand, FPGA-based acceleration solutions have proven to be as close as some high-end GPUs while maintaining a low power consumption; this explains their popularity in this domain [14,17].

However, due to size and memory limits [18], hardware acceleration on embedded reconfigurable devices remains a difficulty, as illustrated by the platform investigation made in [13]. Therefore, several optimization techniques at the hardware level, such as parallel computing, pipeline, or systolic array, should be carefully used in order to meet the constraints.

The authors of [19] suggest ‘DLAU’, an FPGA-based accelerator for large-scale DNNs. The DLAU architecture employs three accelerated pipeline processing units. The hardware deployed on the Xilinx ZYNQ Zedboard platform in conjunction with an ARM Cortex-A9 processor consumes less power. Hardware resources can be consolidated into a single DLAU core that handles all layers, enabling large-scale DNN implementations at the expense of lower throughput.

Maria et al. [20] presented DNN implementations in FPGA employing Stacked Sparse Autoencoders (SSAE) to enable low power architectures for real-time object detection in autonomous systems and robots looking for edge solutions. OpenCL, a programming language for heterogeneous parallel systems, was used to model the accelerator. To categorize the CIFAR-10 color dataset, a Stratix V D5 FPGA was utilized to accommodate a stacked autoencoder. The use of a high-level programming language does not prevent achieving high performance and power consumption efficiency on FPGAs, where 0.357 W and 45 FPS were achieved for a 3072-2000-750-10 SSAE topology.

Coutinho et al. [21] designed an implementation based on Stacked Sparse Autoencoders. Parallel processing elements (PEs) have been used to calculate the basic neuron operation as well as a systolic array technique for streaming DNN weights to enhance the overall throughput. Their systolic network, combined with an entirely hand-written RTL code, makes their solution almost 2.2× faster compared to [20].

It has to be noted that the proposals found in [19,20,21] were about optimization techniques rather than design automation. However, the best results presented in [21] will challenge our proposal, as the resulting DNN implementation is highly optimized.

FPGA-based accelerators take substantially longer to design than software solutions. They demand a high level of electronics skill, especially when it comes to custom optimizations using Hardware Description Languages (HDL). As a result, various efforts have concentrated, in recent years, on specialized frameworks and tools enabling the automatic generation of DNN architecture designs for FPGAs combining customized RTL designs and high-level languages, as detailed in [15,22,23,24,25]. Specific frameworks that offer such design automation will now be reviewed.

Mouselinos et al. [22] presented the ‘TF2FPGA’ framework to inference and accelerate TensorFlow DNNs on FPGAs. Several techniques were used, such as 1-bit input mapping, 8-bit unsigned integer quantization, and a pre-built VHDL library, to optimize the accelerator performance. The weights are extracted from the TensorFlow model and stored as ROM on-chip memory. However, this technique limits the flexibility of the design, as a mandatory rebuild of the whole FPGA architecture must be done if any change on the user application is performed. The experimental results were performed on ZYNQ 7010 SoC.

Mousouliotis et al. [23] created CNN-Grinder, an automated workflow to map a CNN on Low-end-low-cost FPGA ZYNQ 7020 SoC. It includes templates that guide the user through creating, verifying, and converting a part of an algorithm into an HLS definition. The user must define in C/C++ the software application (i.e., the main file) as well as the HLS description for FPGA acceleration using pragmas.

In [15], a high-level design automation framework was presented to enhance the mapping of regular and irregular CNNs models. Their automated design technique, based on Synchronous Data Flow (SDF), allows for fast exploration of architectural alternatives. Within the same power budget, designs implementing this framework performed 6.65× faster than massively parallel GPUs and 2.94× faster than cutting-edge CNN FPGA-based implementations.

Acosta et al. [24] also present a tool that automatically builds customized FPGA-based hardware accelerators for CNN models, which TensorFlow inspires. Using a Graphical User Interface (GUI), the proposed tool allows the user to select the dataset and customize CNN models. MNIST, CIFAR-10, and STL-10 datasets were used to train CNN models. Five CNN models were developed with Tensorflow and compared in that research. The results for the original LeNet-5 design reveal a latency per frame of 1.08 for 32-bit architectures and 0.58 for 16-bit architectures.

Mazouz et al. [25] proposed a design flow for automating FPGA-based reconfigurable CNN models using MATLAB. Indeed, the proposed framework is automated using a workflow technique that allows a designer to get a CNN architecture as well as the option of introducing new latency and space constraints. Via loop reordering, unrolling, and pipelining, the framework automatically generates multiple design spaces to find a trade-off on the latency for resource utilization by varying PEs.

These design frameworks are good examples of the various strategies which can be used to automate and optimize the deployment of hardware accelerators, but they are still not close enough to non-experts in hardware design looking for embedded solutions.

In this paper, we incorporate the previous techniques to create an end-to-end framework for automating optimized DNNs for advanced applications on low-power embedded platforms. Indeed, as presented in the state-of-the-art, we took several advantages of hardware techniques that have been proven effective for DNN implementations, especially pipelining, parallel processing, and systolic array, as discussed in [19,20,21]. In addition, our design methodology supports a front-end user interface to customize the DNN topology and balance the optimization techniques in order to achieve the best trade-off between FPGA architecture performance and hardware limitations. This balancing method does not cover only PEs as detailed in [25], but also the interfacing of each customized IPs (i.e., DNN layers), in order to meet performance requirements (as presented in Section 5.1) and satisfy the variety of communication interfaces available on the platform used. To overcome the barrier that is struggling non-experts (i.e., software developers) to deploy the proposed methodologies, especially those presented in [15,22,23,25], we provide a Python library to define the DNN at the software control layer level in a similar manner as popular frameworks, such as TensorFlow and Keras, as detailed in Listing 2 and Section 6. In addition, we provide a novel approach to generate the FPGA architecture from the Edge by the bias of a harmony communication between the hardware board and the host server or commercial Cloud technologies, e.g., Amazon Web Service or Google, where all the necessary HLS tools are pre-installed and configured. With this method, the user can customize, generate, and deploy DNNs models directly on the Edge without the need of the board’s tools (i.e., Xilinx tools) or any additional commercial tool like MATLAB as in [25]. Based on our knowledge, our work presents the first design automation methodology to deploy DNNs based-FPGA acceleration from Edge-to-Edge. Further explanation details are provided in the following sections.

## 3. General Overview of the Proposed Framework

Most SoCs support a combination of peripherals (e.g., SPI, HDMI, UART, USB) as well as some General Purpose Input/Output pins (GPIO). In the case of PYNQ boards such as Z1, Z2, Ultra96, RFSoC 2x2, etc., additional peripherals can be connected from general-purpose interfaces including Pmods and Arduino via adapters [26]. Besides this adaptability, the ZYNQ 7000 SoC (the integrated chip on the PYNQ board) can offer the best solution by providing hardware acceleration through its programmable part (i.e., FPGA).

**Figure 1:**
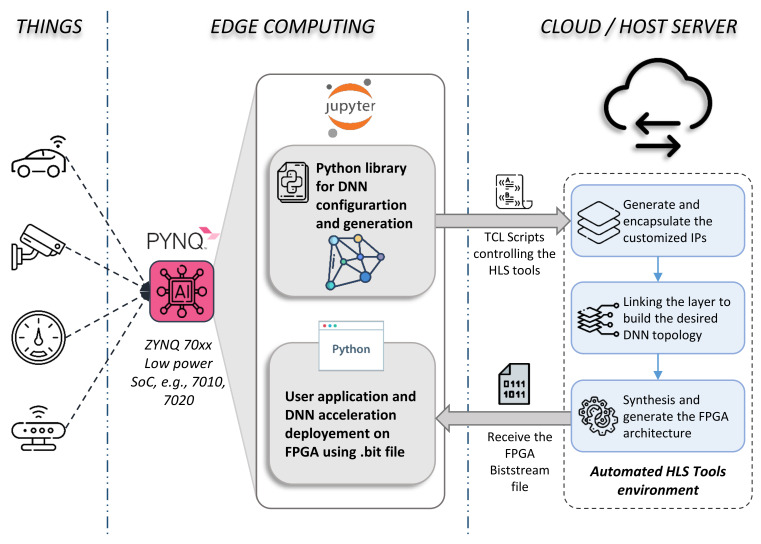
Proposed framework for fully automating the generation and deployment of DNN models based on embedded FPGA acceleration. (**Things**) Sensors are connected to the input ports available on the board, e.g., HDMI, USB, SPI, etc. (**Edge Computing**) is considered as the main SoC chip (embedded FPGA + CPU) driving our fully automated framework. The latter consists of mainly two parts: a Python library to configure, encapsulate, and generate DNN layers, and the user application part where the user can deploy the bitstream of the auto-generated DNN model. (**Cloud/Host Server**) The board is connected to the server where all needed HLS tools to generate such FPGA architecture are pre-installed. The framework sends a TCL file commanding the tools. Once the latter completes their synthesis compilations, the framework gets back the generated bitstream file to configure the FPGA.

Exploiting this compact and low-power embedded system, we present in Figure 1 an overview of our novel Edge-to-Edge framework aimed to fully automate DNN-based FPGA acceleration. The figure presents three main parts depicted into three columns: Things, Edge Computing, and Cloud/Host server on the left, middle, and right column, respectively.

The left column refers to the uncountable peripherals that can easily be connected to the board via the external I/O ports, such as security cameras, microphones, industrial sensors, and actuators. These are treated as the data to be processed by the DNN approach.

Since I/O port IPs are already available and free to use on open source platforms [27], our framework focuses on the most challenging part, which is the DNN acceleration IP. This task is ensured by an automated environment in both Edge and Cloud sides shown on the middle and left columns, respectively. At the Edge, Computing level is considered the SoC board (i.e., PYNQ), where a Linux OS runs on the ARM CPU. All automation processes are controlled via the interactive web application ‘Jupyter Notebook’ pre-installed on the Linux system. At this level, our framework is composed of two sub-Python sections: (1) automate configuration, optimization, and generation of DNN topologies, and (2) automate FPGA bitstream deployment and user application integration, presented with the top and bottom gray squares, respectively. In the first section, the user defines his DNN topology by customizing the layers. The framework generates and stacks layers, creating a unique hardware kernel accelerator (i.e., IP) for each layer. Several optimization parameters, such as pipeline, parallel processing, systolic array, or interface communication, can be applied to ensure a suitable hardware implementation. In the second section, the user will receive the bitstream file to configure the FPGA and the hardware description architecture (i.e., C/C++ dynamic library) once the HLS tools finish compilation. With these files, the user can automate the deployment of the DNN accelerator. In addition, we developed a Python interface to adapt the generated hardware architecture to the user application as described in Section 6.

As HLS tools cannot be used directly on embedded platforms, the compilation process is performed on the host server or commercial clouds such as AWS or Google Cloud, where all HLS tools are pre-installed and configured for automatic compilation. Moreover, third-party DNN IPs can be stored there and deployed when needed. The compilation flow is presented in the right column in Figure 1. In fact, the automation process is driven by a TCL script generated by the framework once the user completes the DNN configuration. The TCL file contains an equivalent “directive” for each layer parameter and the necessary commands to execute the tools. This automation is applied on a C/C++ template specifying the main functionality of layers. More details about each part of the framework are presented in the following sections.

## 4. Framework at the Edge: DNN Configuration and TCL Generation

### 4.1. IP Layer C/C++ Base Template

Starting with a C/C++ template representing the layer to be customized, the proposed framework generates a DNN model FPGA-based acceleration. In order to create such a DNN topology, it generates several layers based on this template by building a specific hardware acceleration IP for each one. Additionally, IPs can be customized with various parameters to maximize performance and meet device constraints. Several optimization parameters, such as pipeline, parallel processing, and interface communication, have been used to customize the hardware implementation of each kernel.

**Figure 2:**
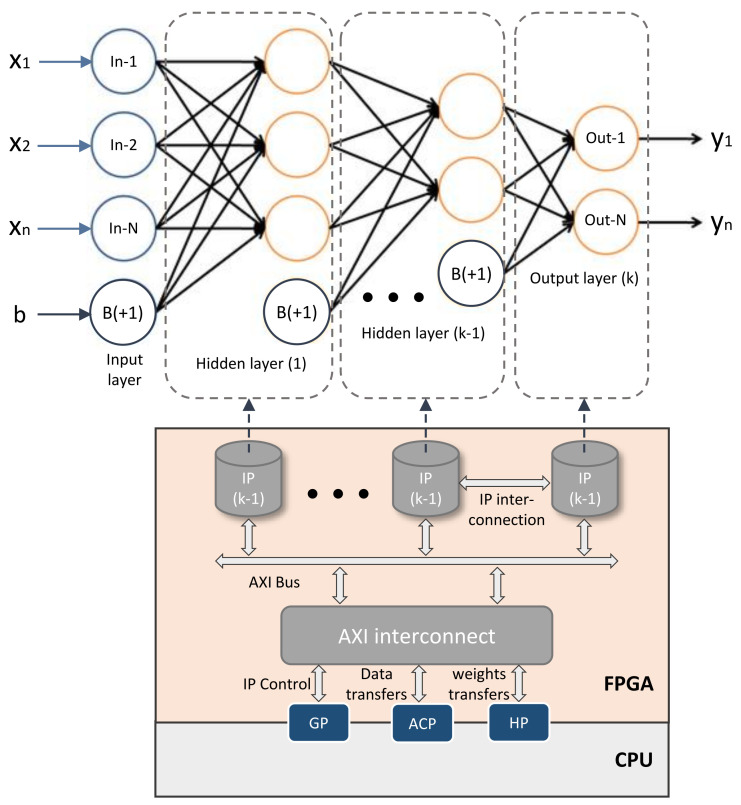
FPGA architecture of the DNN topology. The mathematical representation of such DNN is presented at the top of the figure. Each layer is implemented as a single IP on the FPGA. IPs are connected with an internal bus. The communications between FPGA and CPU are performed via AXI port interfaces (i.e., GP, ACP, HP ports). Data transfers, as well as IPs control, are achieved through AXI bus and AXI interconnects manager.

The C/C++ template represents the mathematical form of each DNN layer as shown in Figure 2. Equation (1) describes the $y_{j}$ output of *j*-th neuron for a given layer. The variable *N* is the number of outputs, i.e., number of neuron. $x_{i}$ is the *i*-th input for the current neuron. *b* value is the bias and $Wb_{j}$ its weight for the *j*-th neuron. $W_{ij}$ is the weight of *i*-th input for *j*-th neuron. Finally, f(∗) represents the activation function.

(1) $$y_{j}=f\left(\sum_{i=1}^{N}\left(x_{i}*W_{ij}\right)+Wb_{j}*b\right).$$

Our C/C++ template is composed of three iteration loops (Listing 1): the first of which is used to multiply the bias by its weight, as depicted in Figure 2. The second loop multiplies and accumulates the input data with DNN weights, while the third is used to produce layer outputs, in other words, the activation function for each output neuron. This organization is done in order to provide flexibility in the layer implementation and full control on the RTL IP version. Section 4.2 explains this in more detail:

**Listing 1.** Pseudo-code for the C/C++ template used for a DNN layer.

*// Multiply the bias value by the weight of j-th neuron*

**for** j = 1 to N_Output

 B[j] = Wb[j] ∗ b


**end**



*// Multiply and accumulate layer weights for all neurons*

**for** i = 1 to N_Input

 **for** j = 1 to N_Output

  X[j] = X[j] + x[i] ∗ W[j]

 **end**


**end**



*// Add weighted bias and weighted neurons and produce output neurons*

*// result via activation function*

**for** j = 1 to N_Output

  y[j] = f(X[j]+B[i]) 
*// activation function of each neuron at k-th layer*


**end**



### 4.2. Python Library: DNN Customization and TCL Script Generation

A couple of HLS directives will be generated automatically to define various level optimizations and the communication interface of each IP. These directives that were chosen consider a highly optimized implementation and appropriate hardware resources in light of the embedded system limitations. However, the user can alter drive them by defining his optimizations to explore other implementation alternatives. Each directive has a different effect on the RTL design synthesized by HLS tools. The main effects can be divided into two categories: the optimization category, where the pipeline and parallel computing can be performed, and the interfacing category containing several AXI interface protocols for different needs.

Directives AXI—Advanced eXtensible Interface [28,29]—define the I/O IP layer communications protocol. AXI is a standard on-chip communication protocol that allows IP to be reused across various modern SoC platforms. Encapsulating the IPs layer with this standard helps to facilitate scalability and compatibility between the generated IPs and different commercial IPs, as well as to other AXI IPs sensors as showed in [27]. For that, there is no lack of adopting our framework with these types of IPs.

In order to allow the user to configure easily, interface and modify the default optimization parameters, a Python library has been developed. In fact, the latter generates a TCL script containing the appropriate HLS directives for each custom IP layer. As a result, the user has control over the hardware implementation and performance. Listing 2 shows our Python library that extends TensorFlow-like functions for FPGA implementation.

Default settings have been provided to simplify configuration. However, the user can provide their own for each layer via the arguments of the Python function. An example of custom parameter modifications related to loops and AXI protocols for the first and second layers are shown in Listing 2. The layer size, however, is a mandatory parameter required for each function, as shown in the last layer declaration.

The Framework automatically sets up and generates the TCL script with the directives once the configuration of layers is complete. When the model.compile() function is called, the framework starts to compile the DNN hardware model according to the TCL script. In fact, the script is sent to the server, where the HLS tools are pre-installed. The automated process will synthesize and encapsulate the IP layers and generate the full FPGA architecture by running the script. Section 5 covers this process in more detail.

**Listing 2.** Python framework to automate the configuration and generation of the DNN model for a specific FPGA.**from** DNN_framework_lib.layers **import** Inputlayer, Hiddenlayer, Outputlayer **from** DNN_framework_lib.models **import**~Sequential *# Configuration* input_layer = Inputlayer(  size=input_size,  loop1="unrolling",  loop3="pipeline_and_unrolling",  input_layer="AXI-MM"  output_layer="AXI-Stream"
)
hidden_layers = [
 Hidden_layer(
  size=layer2_size,
  loop3="pipeline_and_unrolling",
  input_layer="AXI-Stream",
  output_layer="AXI-MM"
 ),

...

 Hiddenlayer(
 size=layerN_size,
 loop1="unrolling"
]
output_layer = Outputlayer(
 size=output_size,
)
model = Sequential(board_name,...)
mode.add(input_layer)
**for** layer **in** hidden_layers:
 model.add(layer)
model.**compile**()

## 5. Framework on the Cloud: Automate HLS Tools for Generating FPGA Architecture

**Figure 3:**
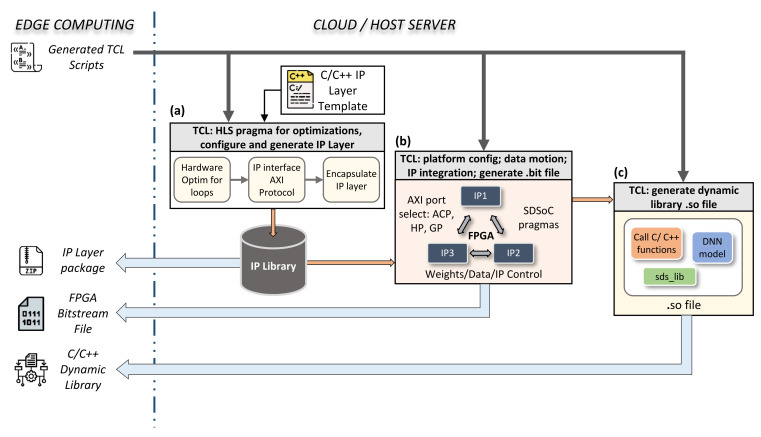
Automated process of HLS tool configuration and synthesis on the Cloud or Host server by the TCL script. The TCL file received from the Edge is responsible for: (**a**) optimizing, interfacing, and generating an IP for each layer based on our C/C++ template; (**b**) specifying the data motion between IPs inside the FPGA and the CPU, configuring AXI ports to be used for each I/O layer, and generating the entire hardware architecture; (**c**) generating the dynamic library to facilitate the control and the execution of different C/C++ function calls from our Python interface.

After the framework generates the TCL script in the first phase of our design flow, the second phase of Cloud or Host server automated IP generation begins. This automated process can be presented as a sequence of three main tasks: generation of the IP layer, the bitstream file, and a dynamic library (i.e., the .so file), as depicted in Figure 3. The TCL script contains all requested layer configurations as detailed in pseudo-TCL script Listing 3. The latter will drive and control the HLS tools in order to provide the appropriate FPGA architecture of such DNN. In this section, we will present these tasks in more detail.

### 5.1. Auto IP Layer Generation

The left square in Figure 3 shows the first task. The task starts with our C/C++ layer template to generate the IP—first, setup, optimization, and synthesis, followed by interfacing the I/O with the appropriate AXI interface protocol. Then, the compiler encapsulates the IP by generating the corresponding RTL design of the latter. It is important to mention that the optimization and interfacing configuration parameters of each layer have an equivalent ‘HLS directive’ as shown in Listing 3.

The directive unroll is used to ensure concurrent computation by generating several instances of the same loop. The internal operands (i.e., $B_{i}$, $X_{j}$, etc.) will be implemented using one block of RAM, which has only two data ports. Therefore, to increase the number of RAM ports which ensure the parallel computing, splitting these arrays into multiple smaller arrays (multiple block RAMs) is mandatory. To do that, we use the directive array_partition for each operand. With the same reasoning, multiple operators (i.e., multiplication, addition, division) instances will be implemented similarly. The latter ensured by using the directive allocation_operation_DSP. This optimization strategy is a powerful method for improving IP throughput. However, on the other hand, parallel computing significantly increases hardware resources, which goes directly against the embedded FPGA limitations. For that, we limit the instance number to only two, as detailed Listing 3.

The directive pipeline has the advantage of reducing the latency of the whole process. As a result, more hardware resources, such as registers, are needed. However, when compared to the unroll directive, which provides parallel execution, the pipeline is less expensive. For this reason, we recommend using the first directive for all layers instead of unroll. Still, the user can explore other configurations to meet the application needs according to the available FPGA resources, as shown in Listing 2.

The second step is interfacing the IP using the AXI bus protocol. The AXI standard has three interface types, each of which is suitable for a specific type of communication. The AXI-Lite requires fewer resources since it allows only one data transfer per transaction. Therefore, we exploited it for IP control and forwarding bias values. The Memory Mapped AXI (AXI-MM) interface is intended for off-chip data exchange, i.e., between IPs and the user application running on the embedded CPU. We use this interface to send the DNN input data; the weights are stored in the DDR memory (off-chip). The last type, the AXI-Stream, is used for inter-IP layer data exchange to ensure high-speed streaming data transactions in a systolic array manner. The systolic array technique bridges the gap between serial and fully parallel architectures [30]. This technique allows serial data to be received and the IP layers to perform their operations asynchronous (the next layer begins to work before the end of the previous one). However, this technique consumes more on-chip memory than the AXI-MM, considering the additional FIFO memory required for data streaming. Listing 3 details the necessary AXI interfacing directives.

**Listing 3**. TCL script that automates HLS tools on the Cloud/Host server.


# Create project, add IP template, and~choose the SoC

create_project DNN

add_files IP_template_cpp

set_top layer_fpga

set_part {ZYNQ_7020}

create_clock -period 10 -name~**default**

 

# Set the appropriate optimization directives according to user configurations

set_directive_allocation -limit 2 -**type** operation DSP_fmul

set_directive_allocation -limit 2 -**type** operation DSP_fdiv

set_directive_allocation -limit 2 -**type** operation DSP_fadd

set_directive_array_partition -factor 2 "layer_1" local_memory

set_directive_unroll "layer_1/loop1"

set_directive_pipeline "layer_1/loop3"

set_directive_unroll "layer_1/loop1"

...

 

# Interfacing the IP layer with the appropriate AXI protocol for each I/O port

set_directive_interface  s_axilite "layer_1"

set_directive_interface  s_axilite "layer_1"

set_directive_interface -mode m_axi -depth size_layer_1 weight_bias

set_directive_interface -mode m_axi -depth size_layer_1 layer_weights

set_directive_interface -mode m_axi -depth size_layer_1 input

set_directive_interface -mode axis layer_1 output

...

 

# Launch synthesis and encapsulate the IP

csynth_design

export_design -rtl vhdl -format~ip_package

 

# Target the board and specify the name of the dynamic library
PLATFORM = PYNQ-z1LIBRARY = lib\_DNN.so
 

# Create an object file for each layer

layer_1_fpga.o: layer_1

layer_2_fpga.o: layer_2

layer_3_fpga.o: layer_3

...

 

# Create C/C++ shared library for the DNN FPGA architecture
{LIBRARY}: layer_1_fpga.o layer_2_fpga.o layer_3_fpga.o~-shared
 

# Generate the bitstream file of the FPGA
create {LIBRARY}.bit


**Figure 4:**
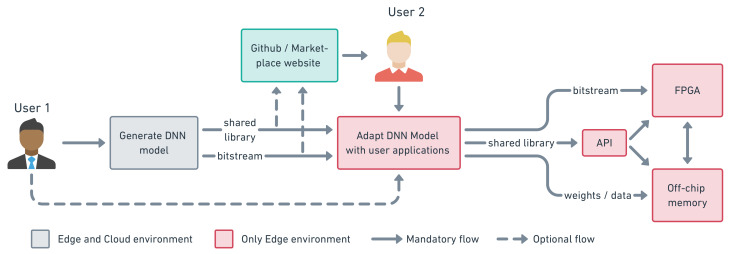
Development workflow, shows an overview of the utilization of the framework.

The final step is to export the resulting RTL design to be used forward by other HLS tools in our design flow. Vivado HLS tools support the compilation, synthesis, encapsulation, and exporting of IP design flow. The generated package can be received from the Cloud and stored on Jupyter repository via the get_IP_package command as explained in Listing 2. Developers can share their own custom architectures on websites such as Github or AWS market-place, as indicated in the workflow of Figure 4 and detailed further in Section 6. This option helps users to save and share all developed architectures as well as create a private DNN library.

### 5.2. Auto IP Layer Integration and Software Control

To build an FPGA-based acceleration architecture for the developed DNN topology, the generated IP layers should be stacked and linked, as shown in the middle box in Figure 3. Since in the previous task all the IPs were perfectly encapsulated with the appropriate interface, there is no obstacle for the compiler to understand the data flow and necessary inter-IP links. The latter also covers linking with CPU interface ports. As our framework is based on the PYNQ Xilinx environment, the target Edge Computing devices are ZYNQ SoC. On them, the software side has three AXI port interfaces with the hardware: General Purpose (AXI_GP), Accelerator Coherency Port (ACP), and High Performance (HP) ports [31].

The AXI GP port is a 32-bit data bus that allows the FPGA and CPU to communicate at low and medium speeds. This port is therefore suitable for IP layer control (i.e., AXI Lite), where only few control commands are required. ACP is a single asynchronous connection between the FPGA and the Snoop Control Unit (SCU) of the CPU. This port is used to ensure coherency between CPU caches and hardware accelerators (i.e., IP layers) within the FPGA. HP interfaces provide FIFO buffers for a burst read/write behavior and high-speed communications. Our framework can instantiate IPs with both ACP and HP AXI, ports for input/output data, and DNN weights, as shown in Figure 2. The HLS tool automatically instantiates an ‘AXI-interconnect’ IP to manage data transactions within the FPGA. In addition to the TCL script, our framework generates a C/C++ header file containing some pragmas driving HLS tools to achieve this configuration.

Once finishing port interfaces’ configurations, the compiler generates a software control driver, a standard object (.o) file, for each IP layer, in order to synchronize communication and data motion of hardware accelerators with the software part (i.e., CPU). The DNN weights and I/O data are passed between the CPU and the accelerator, and the software program will access them after the IP acceleration is completed. Both IPs layers and data motion control are accomplished using the sds_lib library of the SDSoC HLS tool [32]. The latter is used for off-chip (i.e., DDR) memory allocation as well as for the C/C++ shared library, as will be detailed in the following section. It is important to mention that our framework invokes the SDSoC sds++ compiler to accomplish these tasks.

### 5.3. Auto Dynamic Library and FPGA Binary Generation

In the previous section, we described how to integrate and link the IPs. A software control driver, a specific C/C++ library (sds_lib), helps to orchestrate data motions between CPU and FPGA. It is important to remind that our objective is to easily deploy a resulting FPGA-DNN to the Python environment (i.e., Jupyter Notebook) without the need to re-generate a hardware architecture for each new user application. To do that, the framework invokes the compiler to build a shared library (.so) of C/C++ software functions with entry points into the auto generated IPs layer implemented in FPGA. This library includes a program review of all IP caller relationships, as well as the execution procedure of the DNN. As shown, the right box shows in Figure 3 the generated shared library contain the sds_lib library as well. The latter is needed by our framework for the entry point from Python environment, as detailed in Section 6.

Once all compilations are successfully complete, the framework will send the binary FPGA (i.e., .bit file) and the dynamic library (i.e., .so file) files to Edge, where the user can adapt the customized DNN to his application. Some additional configurations to harmony communications between the Cloud/Host server environment and the Edge are presented in Section 6.

## 6. Framework to/from Cloud to Edge: Communication between the Cloud and the Edge Applications

This section describes the communications between the client (i.e., PYNQ on the Edge) and the server (i.e., Cloud). The client side runs on the board. When used by IP developers, the client runtime generates the TCL script and sends it to the server to be executed as described in the pseudo-code Listing 4. The server receives and executes the scripts. In the case of users, as well as for developers, the server will return the resulting hardware binary (.bit) and library (.so) files of the accelerated code. The next step is to upload the (.bit) into the FPGA and create a Python interface for the shared library.

**Listing 4.** Pseudo-code of the function that generates the bitstream (.bit) and the shared libraries (.so) files.

**class** Sequential:

    ...

    **def compile**(self):

        self.generate_tcl_templates()

        sef.send_commands(host="adress_to_server")

        bitstream = self.get_bitstream()

        shared_lib = self.get_SO_library()

        **return** bitstream, shared_lib *#path to the files*



The Host or Cloud server is configured with all the necessary tools to compile customized DNN. A containerized solution using “docker” was developed with all the required configuration to run the tools and facilitate the deployment to the host server or a commercial Cloud platform like AWS or Google Cloud. The runtime communicates with the board via TCP, as the server-side waiting for commands such as receiving, sending data, and executing the TCL script as specified in the pseudo-code Listing 5.

**Listing 5.** Pseudo-code of the server runtime main loop.

**while** True:

    cmd, args = recv_cmd()

    **if** cmd == "get_bitstream":

        send_file(path="path_to_bitstream")

    **elif** cmd == "get_sharedlib":

        send_file(path="path_to_sharedlib")

    **elif** cmd == "exec":

        execute_cmd(args)

    **elif** cmd == "recv":

        recv_data(args)

    ...



The Python API shown in Listing 6 is created using “ctypes”, where we can identify and execute the (.so) inside Python. The framework will wrap all the (.so) functions to abstract the low-level pointer manipulation and memory allocation that need particular alignment. To configure the FPGA, we use the class “Overlay” from the PYNQ library, where it is already pre-installed with the Python package.

**Listing 6.** Pseudo-code of the our Python API for the Model creation.

**from** DNN_framework_lib **import** Sequential

**from** pynq **import** Overlay

**import**~ctypes


 


**class** Sequential:

    ...

    **def** __init__(self, board_name=None):

        self.board_name = board_name

    **def** load_model(self, interface):

        ...

    **def** predict(self, X):

        **return** self.interface.predict(X)

    ...


 


**def** Create_Model(bitstream, shared_library):

    overlay = Overlay(bitstream) *# load bitstream into FPGA*

    shared_lib = ctypes.CDLL(shared_library) *# load lib into memory*

    interface = create_interface(shared_lib) *# adpat C functions to the python*

    model = Sequential().load_model()

    **return** model



From the perspective of the user that only wants to use an already existing architecture, he can import the two generated files (the .bit and .so) into the framework and use them as shown in the pseudo-code Listing 7.

**Listing 7.** Pseudo-code shows the adaptation and utilization of the Model.

**from** DNN_framework_lib **import** ∗

 

model = Create_Model(bitstream, shared_library)

model.predict(X)



Figure 4 describes the possible utilization of the framework. Here, we have two different uses; developer (1) and regular (2), with different possible workflows. The dashed arrows indicate an optional task as opposed to regular arrows that are obligatory ones. Here, the user 1, the IP developer, is responsible for the creation of the DNN architecture and export the generated files in his job or to upload them on some website (e.g., Github, AWS marketplace) to be used by someone else. The user 2, a regular user, on the other side, exemplifies the process of utilizing an already generated architecture; here, this user can download and adapt the existing architecture to his application.

## 7. Experimental Results and Discussion

The synthesis results of a developed IP layer using our automated system are detailed in the first part of this section. The Vivado HLS tools were used to obtain the all performance measurements (synthesis and simulation results). The implementation results of many applied DNN topologies are presented as well. For easy and fair comparison with the state-of-the-art, in our evaluations, we used the MNIST dataset as a case of study [33]. A Xilinx PYNQ open source Python environment [34] is operated on the low cost and low power ZYNQ 7020 SoC board, since our framework’s target domain is Edge Computing and IoT applications. In fact, Edge Computing and real-time embedded systems make extensive use of the above [35,36,37,38,39]. On the PYNQ’s Linux OS image system, a Jupyter Notebook and associated Python package are already configured and installed. In the second part, we compare our design flow with the state-of-the-art and provide a brief discussion about the benefits of our design flow and framework.

### 7.1. Synthesis and Implementation Results

**Figure 5:**
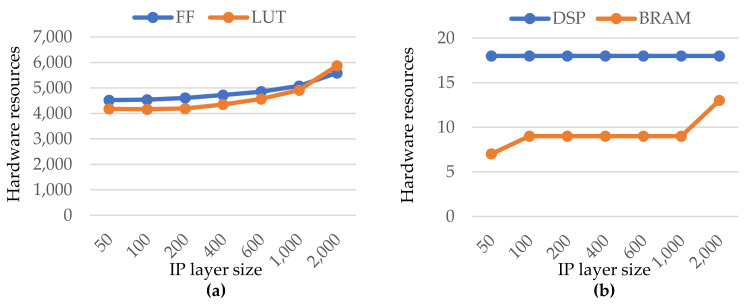
Hardware resources as a function of the IP layer size (i.e., number of neurons). (**a**) blue and orange curves represent the Flip Flop and Look-up Tables units, respectively; (**b**) blue and orange curves represent the DSP and BRAM blocs, respectively.

Figure 5 depicts the hardware resources of a generated custom IP layer using the default directives. The number of Flip-Flops (FF) and Look-up Tables (LUT) consumed as a function of IP size (i.e., the number of neurons) are represented in Figure 5a. The almost linear growth of hardware resources with the IP size can be seen. Given the large variety and number of neuron (from 50 to 2000), the growth is still acceptable. Furthermore, for the largest IP size (2000 neurons), the FF and LUT occupations are less than 6% and 8% of the total available, respectively. The DSP and BRAM block units are represented in Figure 5b. In the covered range of the IP size, both occupations were also quite low. Indeed, as with the prior ones, the utilization percentage is modest, less than 10% and 9%, respectively.

**Figure 6:**
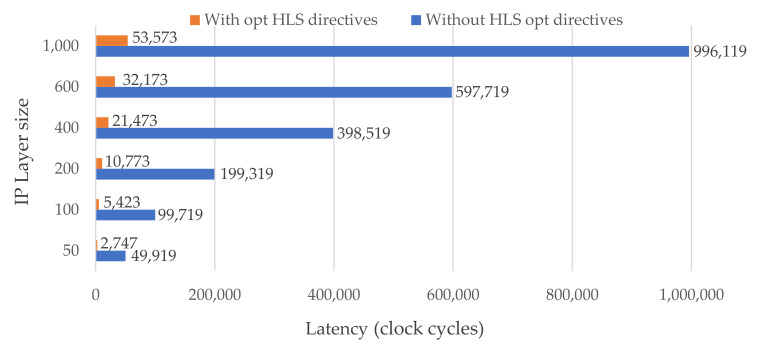
Latency in clock cycles for different IP layer sizes (i.e., number of neurons). The blue and orange bars represent the latency of IP without and with default optimization configuration parameters, respectively.

The latency of the entire IP layer is represented in clock cycles in Figure 6. The values were first obtained from simulations and later confirmed by implementation results. The obtained results with our default optimization directives are shown in orange bars, while the latency without optimization is shown in blue bars. The default directives have a noticeable positive effect. In fact, for a customized IP layer with 50 to 1000 neurons, the number of clock cycles decreases by almost 19×. By adjusting the IP layer configuration parameters as defined in Section 5.1 and Listing 2, the user can easily explore other outcomes suitable for the target FPGA and desired output. The impact of the optimizations and the type of the IP interface on the entire DNN architecture are detailed in Table 1 and Table 2.

To assess design exploration abilities and allow subsequent software version comparisons, Table 1 lists the most prominent topologies auto-generated and implemented by our framework. It is worth noting that the 32-bit floating-point data format is used in the hardware implementation. With this choice, it is indeed very easy to place ourselves in a case where our tool wins on all aspects used for comparison and results discussion. In addition, we could challenge our tool that offers automated optimization versus ‘hand-written’ optimization. In terms of precision, this alternative guarantees a fair comparison with the pure software version. Indeed, the accomplished accuracy for the deployed topologies is sustained between 96.2% and 99.2%, as predicted.

Despite these high quality results, energy consumption remains low, with the smallest topology consuming only 0.26 W and the largest consuming less than 0.43 W. Our architecture not only offers an easy-to-use hardware interface, but also significant acceleration performance, as seen by the speedup results. Table 1 summarizes each accelerated DNN implementation speedup, which is nearly 61× for all alternative topologies as opposed to the pure software variant running on the embedded CPU Cortex A9 Dual-core, at up to 1 GHz. The speedup seems to be relatively high because, in the CPU code, there are many sub-optimal operations. In fact, a simple function like calling a math function from an external library takes at least a thousand instructions since it needs to go through the dynamic loader to find the library, then allocate if needed and load into the memory, then calculate the addresses set up the stack, jump to the global offset table, and finally run the code that we need. In contrast, the same function in the FPGA could be executed directly in a few hundred cycles.

Moreover, the achieved performance results, especially those of power consumption, show that the proposed design flow allows for meeting low power requirements. This is critical for IoT applications exploiting the Edge Computing, since the increasing computational effort affects battery lifetime of many mobile devices [5].

### 7.2. State-of-the-Art Comparison and Discussion

We chose some reference DNN solutions to evaluate the position of our framework in the context of hardware acceleration, as well as to assess its embedded capabilities. Meanwhile, we have reported the analysis of three alternative implementations for the same DNN topology, the 784-100-50-10, while balancing between the flexible parameters (i.e., pipeline and parallel optimizations; and AXI interfaces) as detailed in Listing 2, in order to assess the optimization impact on the performance results. Table 2 summarizes the findings of state-of-the-art works for the using MNIST database. Topology, clock frequency, throughput in Frames per second (FPS), and power consumption are some of the implementation and performance parameters mentioned in the table. Since not all topologies are equally implemented, we also include the data type, topology complexity presented in the number DNN parameters, and the throughput in terms of Million parameters per second (Mps). The latter may aid in understanding the correlation between the throughput and the topology complexity.

Our first alternative provides an implementation devoid of any kind of optimization. This is expressed in the performance result, where the throughput record just 1.59 Mps and 19 FPS, the lowest possible results. We adopted a pipeline and parallel optimizations strategy in the second alternative. When compared to the first alternative, the throughput increases up to 9.917 Mps and 118 FPS, which is more than 6× faster. The third alternative implementation is the best strategy, as we used an AXI-Stream at the inter-layer communication protocol in addition to the previous optimizations of the second alternative in order to perform a systolic array technique. As compared to the first and second implementation alternatives, the overall results increased by 61.3× and 9.8×, respectively. As less AXI-MM are used, the power consumption was reduced by almost 100 mW, from 0.49 W–0.38 W.

Comparing with the state-of-the-art, the authors in [21] propose an SSAE optimized at the low level, custom RTL, to achieve the best process efficiency in terms of throughput and power consumption. Indeed, this proposal has the highest throughput and uses the least amount of resources. Nevertheless, they use 12-bit data types to achieve this efficiency, resulting in lower accuracy. However, since this work is aimed at high-performance computing, the proposal was deployed using high-end powerful boards like the Virtex-6. The latter does not present a perfect solution for IoT applications looking to its high price, which costs more than 1821 US Dollars (USD) according to the “Digikey” website [40].

Mazouz et al. in [25] propose a design flow for automating FPGA-based reconfigurable DNN models. For the MNIST classifier, they introduced many topologies. Table 2 depicts the chosen topology 1-2-4, with three hidden convolutional layers and seven filters, where the total parameters are almost 41 k. Notice that the implementation was done in much larger ZYNQ, the ZYNQ 7100, which costs around $4043 USD, but just using 16-bit fixed-point data values instead of 32-bit, which slightly reduces the accuracy ( 98.6 ). However, the throughput do not illustrate any of this (still low at 526 FPS). Furthermore, although the power consumption is not high, the efficiency ( 26.35 /Mps) is somehow.

Rivera-Acosta et al. [24] provided a GUI for generating several CNN topologies automatically using only RTL templates. For the outcome assessment, they have used Cyclone IV FPGA. They achieved a high throughput of 925 FPS with their LeNet-5 topology and 32-bit data types. However, the throughput in Mps (55.5 Mps) is relatively low with a limited number of parameters opposed to others. They did not, however, present any details related to power consumption.

Even with high-level synthesis and 32-bit data types, our automated approach delivered considerably outstanding results in terms of throughput, both in FPS and Mps, compared to [21,24]. Furthermore, the power and efficiency figures are very similar to those obtained using optimized RTL models as in [21]. Additionally, it is important to note that our implementations were done using a low cost SoC-based FPGA accelerator, the ZYNQ 7020 ($125 [40]), which presents a better solution for IoT applications compared to [24,25], where ZYNQ 7100 ($4043 USD) and Cyclone IV ($340 USD) are used, respectively. With this analysis, our framework resulting in the best balance of throughput, power consumption, efficiency, and system cost.

**Figure 7:**
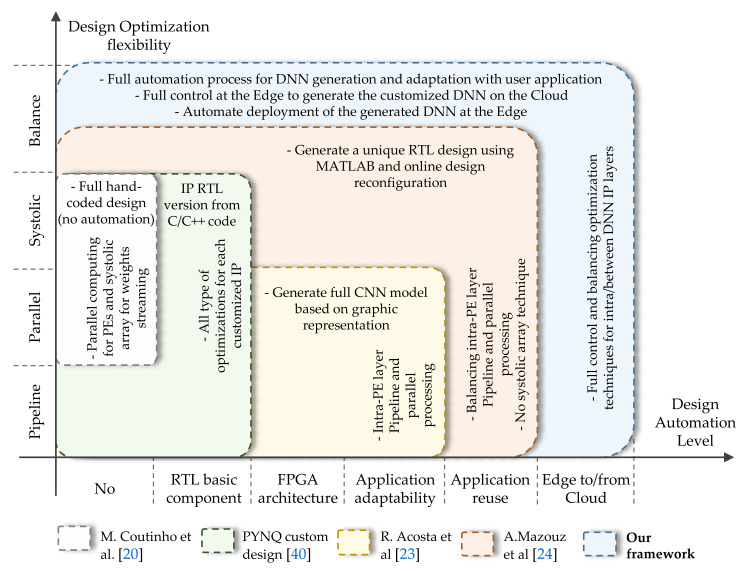
A comparison between our framework features and the state of art works. The *x*-axis represents the level of DNN automate generation where the *y*-axis represents the optimization that can be performed by each proposed work.

Figure 7 illustrates our framework features related to hardware optimization flexibility (*y*-axis) and the level of automation (*x*-axis) compared with the state-of-the-art works. Each colored square represents a specific work or methodology, where its spread area in the plane *x*-*y* reflects the work’s capabilities. Our framework has the largest area colored in blue.

In Figure 7, the gray square represents the work of Coutinho et al. [21]. They have used parallel Processing Elements (PEs) to calculate the basic neuron operation as well as a systolic array technique for streaming DNN weights to enhance the overall throughout. This work gives the best results in terms of power consumption and throughput since it uses custom RTL PEs. However, this work does not offer any kind of design automation. The green square depicts the basic PYNQ design methodology [41]. The user has to design an “overlay”, the FPGA architecture (in our design terminology the bitstream), and the project block diagram, a TCL file. To build a specific DNN model, the user can start with the overall DNN model topology, or by layers (the IP layer in our framework) and then create the FPGA architecture by doing the necessary connections between IPs and the processing software (i.e., the embedded CPU). In this methodology, all types of optimization (pipeline, parallel, systolic array, etc.) can be done at this design level. Using the VIVADO tool, the RTL design of each IP can be automatically generated starting from C/C++ code. Still, the user has to develop a specific Python interface in order to deploy and adapt the FPGA DNN accelerator with the Python application. In the work of Acosta et al. [24], presented in the yellow square, a full CNN model can be generated based on a graphic representation. The optimizations were performed at the intra-layer level, where the PEs are multiplied to ensure parallel and pipeline computing. There is no optimization ability for transfer data between layers, such as systolic array or pipeline. For this purpose, the whole process should be done for each new application, starting from training a CNN model to the generated FPGA architecture design. The last compared work is presented in the orange square, where Mazouz et al. [25] provide a framework to generate a unique RTL design for custom DNN models using MATLAB. As in [24], a pipeline and parallel processing at the PEs level was proposed in order to minimize the latency of the processing layers. They propose a flexible optimization level, where the user can define the number of PEs running in parallel. However, no optimization between layers was proposed. The resulting unique RTL design can be online implemented on the target FPGA.

In our framework, we offer the highest level in both design optimization flexibility and design automation, generation, and deployment, compared to the previous works. In fact, pipeline and parallel optimization were adopted at the intra-layer lavel as [21,24,25,41] and a systolic array technique for data streaming as [21,41]. Furthermore, optimization balance is proposed as in [25], in addition to a systolic array for data streaming between layers by changing their interface type as described in Section 5.1 and Listing 2. In this manner, our design flow offers the opportunity to generate a unique circuit tailored for each application, in order to meet the user necessity and achieve better trade-off between performance and hardware constraints. As presented in Figure 7, the whole process of our framework is fully automated starting from basic RTL components (i.e., IP layers) to the adaptability and re-usability with user applications as detailed in Section 5.3 and Section 6. Besides what is detailed on the state-of-the-art, we propose a novel Edge-to-Edge methodology to fully control, customize, generate, and deploy DNN models from Edge. This methodology is ensured by a harmony communication between the Python interface running on the Edge and the HLS tools pre-installed on the Cloud or host server as shown in Figure 1.

### 7.3. Enhancement of Design Flow for IoT Applications-Based Edge Computing

The proposed framework enables the design to be done in a similar way to “pure software” neural network applications using the popular Python interface. This feature satisfies one challenge facing IoT applications-based edge computing, the “availability”, a concept developed in [42] about the successful deployment of edge computing in IoT. The “availability” includes three parameters, i.e., the mean time between failure, the failure probability, and the mean time to recovery. Using the proposed edge-to-edge framework, one can easily optimize the time to recovery thanks to the Python interface as well as the fully automated generation process. In addition, the workflow of our framework makes it possible to quickly recover the original generated architecture stored on the cloud without any additional engineering work, as shown in Figure 4. It is also unique to edge-to-edge applications as the usual design flow requires gathering many different tools and methodologies for the full development, whereas the proposed design flow unifies the hardware (i.e., the acceleration) and the software under the same interface, and this will automatically enhance the “availability” of the application.

The second challenge facing edge application is the adaptability of the architecture, where standard protocols and interfaces are required, as mentioned in [5,42]. In fact, different devices and sensors connect and communicate with one another and with the edge server via communication protocols. Considering that different vendors manufacture different devices in the IoT environment, standard protocols and interfaces should be developed to enable communication among these heterogeneous devices [42]. In our design flow, we respond to this obligation by using only the well-known AXI interface protocol, where it is used for all generated IPs and the entire FPGA architecture, as mentioned in Section 4.2.

The third feature enabled by the framework is the possibility of configuring the hardware for each layer, where we can add and balance between loop unrolling, pipeline, and other hardware accelerating methods in order to meet hardware resources’ limitations. This feature helps deploy DNN architectures in a wide range of FPGAs, starting from the minimal resources and power consumption devices to the latest powerful ones dedicated to edge computing.

## 8. Conclusions

In this paper, we presented our novel design flow of DNN based embedded FPGA acceleration on the Edge for low power and IoT systems. With little to no previous FPGA or hardware design experience, developers of hardware-accelerated DNNs may use a familiar Python-centric programming workflow to take advantage of FPGA acceleration. Our design flow offers the opportunity to generate a unique circuit tailored for each Python application in order to meet the user’s necessity.

Besides the automation of our design methodology, the latter is fully deployed on the Edge. For that, the user starts with a Python interface where he customizes the hardware implementation. A TCL script is generated automatically to drive the HLS tools while respecting the user’s customization. Our novel approach is to command the tools automatically from the Edge. In fact, a runtime is executed on both sides, Edge and Host server or commercial Cloud, to harmonize the communications and transfer files. Once the HLS tools finish compilations on the Cloud, they send back the necessary files for FPGA configuration and its software control. An API wraps the received files in order to adapt and re-use the generated DNN architecture easily. The user can share the designed architecture in a public website (e.g., Github) or marketplace (e.g., AWS marketplace) as well.

By using only a dedicated board, it can be possible now to generate with our framework customized DNNs totally on the Edge without the need for private tools. Based on our knowledge, our work presents the first Edge-to-Edge automation framework for DNN-based FPGA acceleration. The state-of-the-art comparison shows that our framework provides the best trade-off between the mandatory IoT criteria abstracted on power consumption, low cost, and high throughput.

The framework has been extensively tested by using a scenario of handwritten digit recognition, the training behind it made on the famous MNIST database. This can lead to the development of a full edge application, such as the automatic recognition of license plates or automatic online filling of handwritten bank transfers.

Our new Edge-to-Edge environment can integrate pure FPGAs design-flow to automate hardware implementation on another boards, other SoCs, and other brands by simply adopting a unique TCL template for each commercial tool-chain. Besides what we mentioned earlier, our future work is developing new IP templates that cover other DNN layer types to generate more popular topologies like CNNs, RNNs, and GANs, among others.

## Figures and Tables

**Table 1:** Performance achieved with the DNN topologies auto-generated and implemented on a ‘Zynq 7020’ board with the proposed framework, for a frequency of 100 MHz, tested on the MNIST database.

Topology	Throughput		Speed-Up	Accuracy (%)	Power (W)
	CPU-Only	CPU + FPGA			
784-32-32-10	60	3636	60.6×	96.2	0.266
784-100-50-10	19	1160	61.05×	99.2	0.380
784-100-50-20-10	19	1153	60.68×	99.2	0.430

**Table 2:** Comparison of performance between state-of-the-art DNN topologies and three implementation alternatives auto-generated with the proposed framework, at a frequency of 100 (information not available for [25]). The mark (*) means: Higher is better. The mark (**) means: Lower is better.

Work	Topology	Chip	Data (Bit, Type)	Parameters (Number)	Accuracy (%)	Throughput		Power (W)	Power/Throughput (mW/Mps) **
						(FPS)	(Mps) *		
[21]	784-100-50-10	Virtex 6	12, fixed	84.05 k	93.3%	1250	105.062	0.3	2.855
[25]	1-2-4	Zynq 7100	16, fixed	41.71 k	98.6%	526	21.934	0.578	26.35
[24]	LeNet-5	Cyclone IV	32, float	60 k	-	925	55.5	-	-
**Ours, #1**	784-100-50-10	Zynq 7020	32, float	84.05 k	**99.2%**	**19**	**1.59**	**0.478**	**300.6**
**Ours, #2**	784-100-50-10	Zynq 7020	32, float	84.05 k	**99.2%**	**118**	**9.917**	**0.49**	**49.4**
**Ours, #3**	784-100-50-10	Zynq 7020	32, float	84.05 k	**99.2%**	**1160**	**97.498**	**0.38**	**3.89**

## Data Availability

Data available in a publicly accessible repository that does not issue DOIs. Publicly available datasets were analyzed in this study. This data can be found here: https://github.com/Tarekro/AutoPy2Fpga (accessed on 18 August 2021).

## Abbreviations

The following abbreviations are used in this manuscript:

AI	Artificial Intelligence
API	Application Programming Interface
AXI	Advanced eXtensible Interface
CPU	Central Processing Unit
DL	Deep Learning
DNN	Deep Neural Network
FPS	Frames Per Second
FF	Flip-Flop
FPGA	Field Programmable Gate Array
HLS	High-Level Synthesis
IoT	Internet of Things
IP	Intellectual Property
LUT	Look-Up Table
Mps	Million parameters per second (DNN parameters)
USD	US Dollars
RTL	Register Transfer Level
SoC	System-on-Chip
TCL	Tool Command Language
